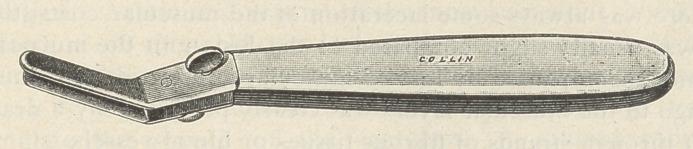# A Chondrotome for Cranioplasty

**Published:** 1917-11

**Authors:** 


**A Chondrotome for Cranioplasty.** By A. Gosset. Trans. and abstracted from the *Bulletins et Mémoires de la Société de Chirurgie de Paris*, Feb. 29, 1916.

The author describes an instrument invented by him for cutting cartilage without destroying the continuity of the portion left. It is a kind of bistoury with a protecting blade fixed above the cutting blade in such a manner as to permit the cut to be made at the desired depth but no farther. The blades are slightly deflected from the handle to permit of easy operation. The author has been able, by means of this instrument, to cut cartilage grafts in one piece large enough to fill a gap 9 cm. in length and 5 cm. in breadth. He recommends the measurement of the bone cavity, when it has been laid bare, by means of a cloth compress. From this, the portion of the cartilage to be cut can be exactly marked in advance, and only enough taken to fill the cavity exactly. In cutting, a leeway of about 2 mm. should be allowed beyond the limits of the model. The cartilage may then be trimmed so as to fit exactly the bony cavity. The side with the perichondrium should be placed towards the brain. The instrument allows a thickness of 3 mm. to be cut, which is just sufficient for rigidity and protection. The author thinks it preferable to employ a single piece of cartilage for the grafts rather than several fragments as is commonly the practice. This is generally possible when his knife is used for the cutting. He claims for it greater rapidity in performing the operation.

## Figures and Tables

**Figure f1:**